# Benefit of Adjuvant Mesenchymal Stem Cell Transplantation to Critical-Sized Peripheral Nerve Defect Repair: A Systematic Review and Meta-Analysis of Preclinical Studies

**DOI:** 10.3390/jcm12041306

**Published:** 2023-02-07

**Authors:** Martin Aman, Matthias Schulte, Yu Li, Benjamin Thomas, Simeon Daeschler, Maximilian Mayrhofer-Schmid, Ulrich Kneser, Leila Harhaus, Arne Boecker

**Affiliations:** Department of Hand, Plastic and Reconstructive Surgery, Burn Center, BG Trauma Center Ludwigshafen, Department of Hand- and Plastic Surgery, University of Heidelberg, Ludwig-Guttmann-Str. 13, 67071 Ludwigshafen, Germany

**Keywords:** peripheral nerve, nerve injury, trauma, stem cell, MSC, nerve reconstruction

## Abstract

Critically sized nerve defects cause devastating life-long disabilities and require interposition for reconstruction. Additional local application of mesenchymal stem cells (MSCs) is considered promising to enhance peripheral nerve regeneration. To better understand the role of MSCs in peripheral nerve reconstruction, we performed a systematic review and meta-analysis of the effects of MSCs on critically sized segment nerve defects in preclinical studies. 5146 articles were screened following PRISMA guidelines using PubMed and Web of Science. A total of 27 preclinical studies (n = 722 rats) were included in the meta-analysis. The mean difference or the standardized mean difference with 95% confidence intervals for motor function, conduction velocity, and histomorphological parameters of nerve regeneration, as well as the degree of muscle atrophy, was compared in rats with critically sized defects and autologous nerve reconstruction treated with or without MSCs. The co-transplantation of MSCs increased the sciatic functional index (3.93, 95% CI 2.62 to 5.24, *p* < 0.00001) and nerve conduction velocity recovery (1.49, 95% CI 1.13 to 1.84, *p* = 0.009), decreased the atrophy of targeted muscles (gastrocnemius: 0.63, 95% CI 0.29 to 0.97 *p* = 0.004; triceps surae: 0.08, 95% CI 0.06 to 0.10 *p* = 0.71), and promoted the regeneration of injured axons (axon number: 1.10, 95% CI 0.78 to 1.42, *p* < 0.00001; myelin sheath thickness: 0.15, 95% CI 0.12 to 0.17, *p* = 0.28). Reconstruction of critically sized peripheral nerve defects is often hindered by impaired postoperative regeneration, especially in defects that require an autologous nerve graft. This meta-analysis indicates that additional application of MSC can enhance postoperative peripheral nerve regeneration in rats. Based on the promising results in vivo experiments, further studies are needed to demonstrate potential clinical benefits.

## 1. Introduction

In a European Level A Trauma Center with 110,667 patients treated, 5026 were identified with peripheral nerve injury [1]. Taking economic impact and impairment of quality of life into consideration, peripheral nerve injuries have a tremendous impact on clinical practice. For this reason, early reconstructive strategies are of utmost importance to promote fast recovery. With many reconstructive strategies available, tension-free reconstruction of the peripheral nerve in its physiologic anatomy is considered best for early recovery. If tension-free coaptation is not feasible due to a critical-sized defect, interposition of the injured nerve with expendable donor nerves or conduits is required [2]. Many different options for the bridging of critical-sized nerve defects have been explored and described in the literature; however, the gold standard today remains reconstruction with autologous nerve grafts [3]. With only a limited amount of available donor nerves for grafting, ideal conditions for recovery are essential and different strategies for refinement have been explored [4].

There are several alternatives to autografts for bridging critical-sized nerve defects, including autologous grafts from non-nerve tissue, hollow or filled conduits from both biological and synthetic materials, and decellularized allografts [3,4].

One strategy for refining these techniques is adding or using materials, cells, or other biological agents with an inherent positive influence on the regeneration of peripheral nerves. Mesenchymal stem cells (MSCs) are cells with the ability to self-renewal and differentiate into adult cells. MSCs are thought to be a viable new treatment for extended nerve defects because of their advantageous effects on promoting axonal regeneration [5,6]. The positive influence of MSCs on peripheral nerve regeneration is hypothesized to be rooted in multiple aspects of their biological characteristics, such as factor secretion, immunomodulation, and their ability for potential further differentiation into Schwann-like cells [5,6,7]. MSCs can secrete a broad repertoire of bioactive factors, including cytokines, growth factors, microRNAs, proteasomes, and exosomes, which can enhance angiogenesis, stimulate axonal regeneration, and prevent nerve tissue from excessive inflammation by depressing overaggressive activation of T-lymphocytes and natural killer cells [8]. In addition, MSCs can be readily expanded in vitro and have low immunogenicity, making their large-scale clinical application possible [9].

Currently, the therapeutic effects of MSCs adjuvantly used to improve different strategies for bridging nerve gaps have only been examined in preclinical animal models. Aiming to synthesize the available preclinical evidence, we present a systematic literature review and meta-analysis of the reported effects and potential clinical application of MSCs in the repair of large peripheral nerve defects [9].

## 2. Methods

This meta-analysis was conducted according to the Preferred Reporting Items for Systematic Review and Meta-Analysis guidelines (PRISMA) [10].

### 2.1. Sources and Search Strategy

Two researchers independently screened the articles based on the inclusion criteria shown in Table 1, and another researcher resolved any discrepancies. Searching for the preclinical studies was completed on 12 February 2022 using PubMed and Web of Science. The search terms were (((peripheral nerve defect) OR (peripheral nerve injury)) OR (peripheral nerve regeneration)) AND ((((mesenchymal stem cell) OR (stem cell)) OR (mesenchymal stromal cell)) OR (stromal cell)).

### 2.2. Data Extraction

Data extraction was performed by two independent researchers, and any discrepancies were resolved by a third researcher. When the data were presented only through graphs, it was extracted with GetData Graph Digitizer software (Version 2.26, http://getdata-graph-digitizer.com/, accessed on 9 April 2022).

### 2.3. Statistical Analysis

The Cochrane statistical software Review Manager 5.3 (Cochrane Collaboration, Copenhagen, the Nordic Cochrane Centre) was used for data analysis and graphs preparation. For continuous outcomes, the effect size was calculated using the difference in means (MD) or the standardized mean difference (SMD) with a 95% confidence interval (CI) in a fixed effect analysis model. When different scales were used to measure the same outcome, we calculated the SMD; otherwise, the MD was calculated.

## 3. Results

### 3.1. Search Results and Study Characteristics

**Figure 1:**
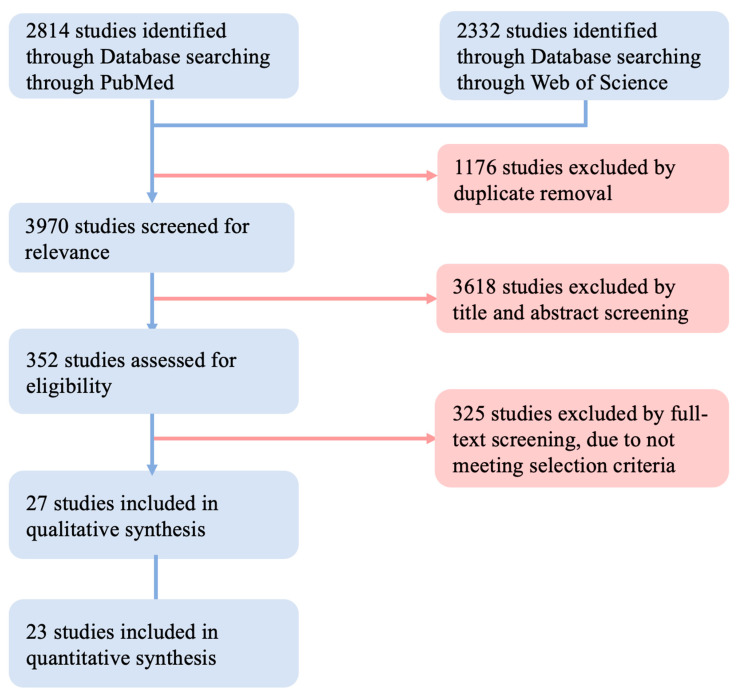
The related studies were searched and included according to the PRISMA guidelines.

The study selection process is summarized in Figure 1.

**Figure 2:**
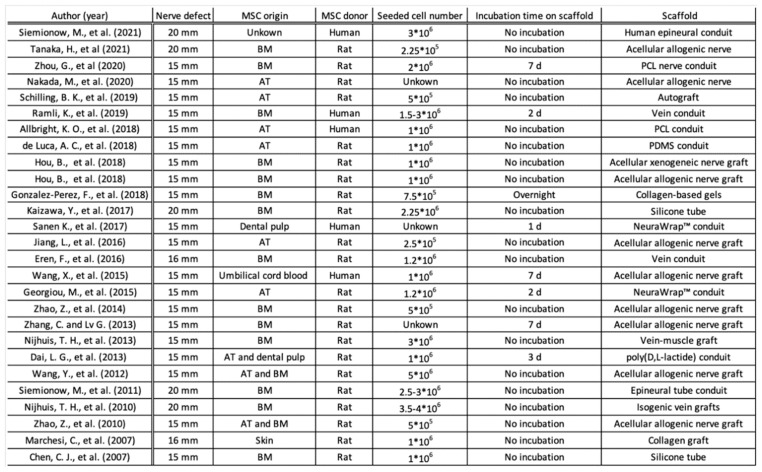
Characteristics of the included studies [5,11,12,13,14,15,16,17,18,19,20,21,22,23,24,25,26,27,28,29,30,31,32,33,34,35,36]. Abbr: AT = adipose tissue, BM = bone marrow, PCL = poly(ε-caprolactone), PDMS = poly-dimethylsiloxane.

A total of 2814 articles were retrieved from PubMed, while 2332 publications were obtained from Web of Science. A total of 1176 duplicates were excluded. A total of 3618 articles were excluded by title and abstract screening. The eligibility of the remaining 352 articles was assessed by full-text screening. A total of 27 preclinical studies with a total of 722 animals were included in the meta-analysis. Their publication date ranged from 2007 to 2021. The characteristics of each study are summarized in Figure 2. All included studies were published in either English or Chinese and used the standard rat sciatic nerve model with defects ranging from 15 mm to 20 mm [5,11,12,13,14,15,16,17,18,19,20,21,22,23,24,25,26,27,28,29,30,31,32,33,34,35,36]. A total of 18.5% of the included studies used human (i.e., xenografted) MSCs, whereas 81.5% used murine (i.e., autografted) MSCs. The MSCs were sourced from bone marrow (51.4% of the 35 treatment groups), adipose tissue (37.1%), dental pulp (5.7%), umbilical cord (2.9%), and skin (2.9%). Only 1 study used autologous nerve grafts, while 12 studies applied acellular nerve scaffolds for nerve defect bridging. The acellular nerve scaffolds in 10 studies were derived from allogeneic donors; acellular xenogeneic nerve scaffolds made of human sciatic nerve and rabbit tibial nerve were used in 2 studies. The nerve conduits used were based on vein, muscle, silicon, collagen, or polymeric materials. Observation times ranged between 4 and 24 weeks, and no dropouts were reported due to the surgery. As axon number varies greatly from the proximal to the distal in nerve defects, meta-analysis was only performed on studies in which the data were obtained from the middle or distal portion of the nerve defect (25 studies included in qualitative synthesis; 22 studies included in the quantitative synthesis of the axon number).

### 3.2. Quality of Included Studies

**Figure 3:**
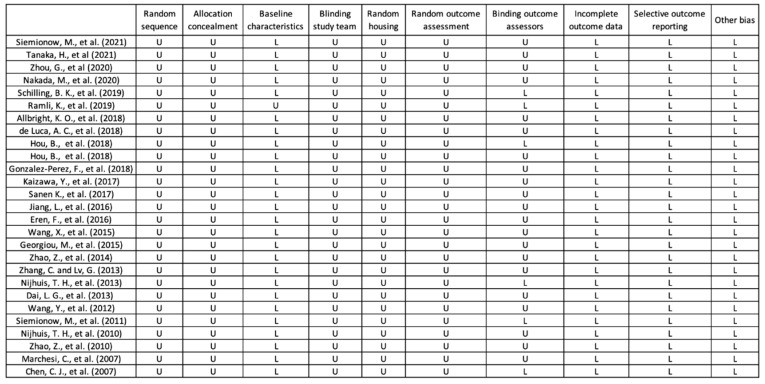
Results of the bias assessment using SYRCLE’s risk of bias tool [5,11,12,13,14,15,16,17,18,19,20,21,22,23,24,25,26,27,28,29,30,31,32,33,34,35,36]. Abbr: L = low, U = unknown.

The quality of studies was assessed using SYRCLE’s risk of bias tool. The results are shown in Figure 3. Most studies mentioned random sequence generation; however, none described a detailed method. Furthermore, only six studies indicated blinding of outcome assessors [12,19,24,26,28,29]. In addition, no descriptions of allocation concealment, blinding of the study team, or random housing were found in any of the studies. Baseline characteristics were well illustrated in all studies except the one by Ramli et al. [26]. Risks of incomplete outcome data or selective outcome reporting appeared to be low in all studies. No other sources of bias were observed.

### 3.3. Effects of Interventions

Six indicators were used to study effect sizes in this meta-analysis: sciatic functional index (SFI), nerve conduction velocity, the weight of the gastrocnemius and triceps surae muscles, axon number, and myelin sheath thickness.

**Figure 4:**
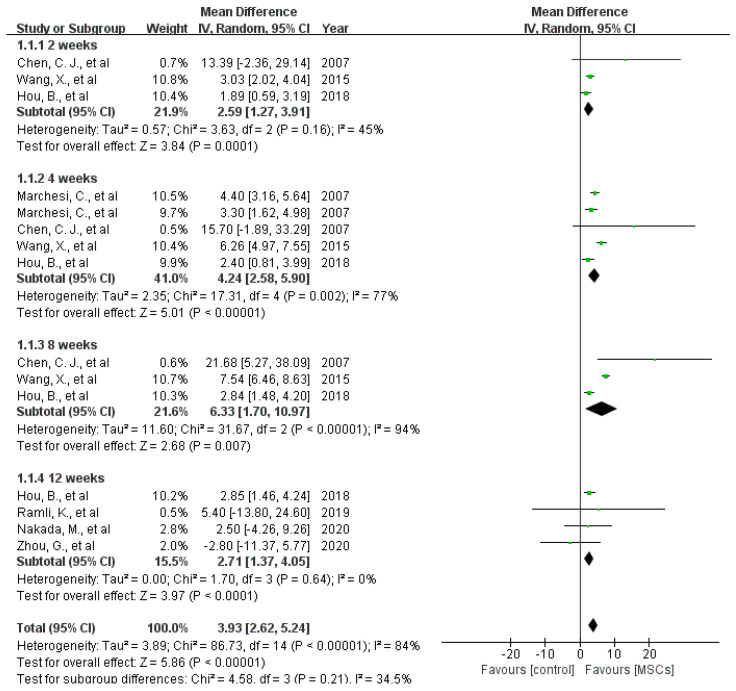
The forest plot shows the effect of MSCs on the sciatic functional index 2 weeks, 4 weeks, and 12 weeks after surgery [5,11,12,13,14,15,16,17,18,19,20,21,22,23,24,25,26,27,28,29,30,31,32,33,34,35,36]. Plots on the right-hand side indicate favoring MSCs or MSCs-loaded scaffolds, while plots on the left side indicate favoring placebo-control treatment or blank scaffolds.

**Figure 5:**
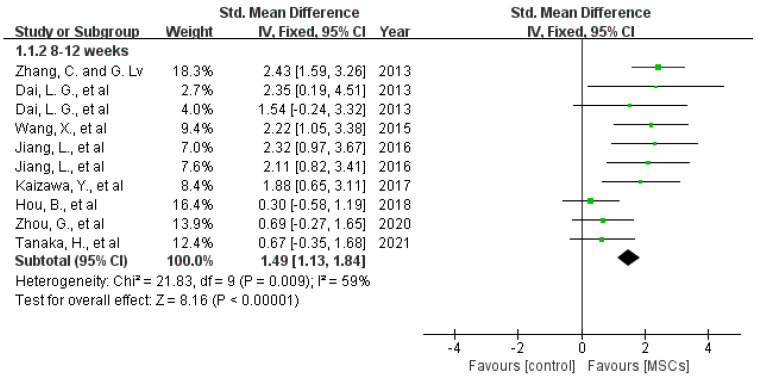
Meta-analysis of the effects of MSCs on nerve conduction velocity 8–12 weeks after implantation [5,11,12,13,14,15,16,17,18,19,20,21,22,23,24,25,26,27,28,29,30,31,32,33,34,35,36]. Plots on the right-hand side indicate favoring MSCs or MSCs-loaded scaffolds, while plots on the left side indicate favoring placebo-control treatment or blank scaffolds.

Nerve function: Eight included studies assessed the motor function by comparing SFI. However, the data in the research paper by Dai et al. could not be extracted from the graphs [12,18,22,23,28,31,36]. Meta-analysis of the remaining seven studies showed that treatment with MSCs increased the SFI score in rats at 2 weeks (difference in means [37], 95% confidence interval [15], fixed effect: 2.59 (1.27 to 3.91), I^2^ = 45%, 61 rats), at 4 weeks (difference in means [38], 95% confidence interval [15], fixed effect: 4.24 (2.58 to 5.9), I^2^ = 77%, 121 rats), 8 weeks (difference in means [38], 95% confidence interval [15], fixed effect: 6.33 (1.7 to 10.94), I^2^ = 94%, 61 rats) and 12 weeks (difference in means [38], 95% confidence interval [15], fixed effect: 2.71 (1.37 to 4.05), I^2^ = 0%, 52 rats) after surgery (see Figure 4). Additionally, sciatic nerve conduction velocity was evaluated in eight studies. All of them indicated that MSCs promoted nerve conduction velocity after 8 weeks (see Figure 5) (difference in standard means (SMD), 95% confidence interval [15], fixed effect: 1.49 (1.13 to 1.84), I^2^ = 59%, 178 rats) [13,18,20,21,30,31,33,36].

**Figure 6:**
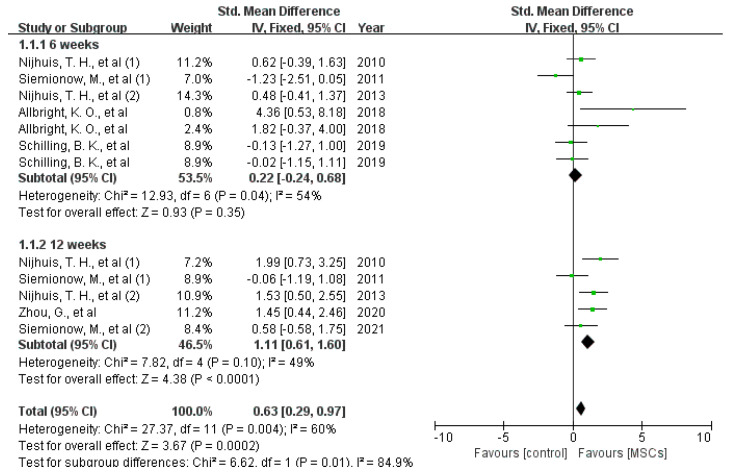
Meta-analysis of the weight of gastrocnemius muscles 6 weeks and 12 weeks after surgery [5,11,12,13,14,15,16,17,18,19,20,21,22,23,24,25,26,27,28,29,30,31,32,33,34,35,36]. Plots on the right-hand side indicate favoring MSCs or MSCs-loaded scaffolds, while plots on the left side indicate favoring placebo-control treatment or blank scaffolds.

Muscle weight: The gastrocnemius and triceps surae muscles are controlled and nourished in dependence on their activation by the sciatic nerve. The weight of both muscles, therefore, indirectly reflects nerve function. Five studies by four research teams reported the effects of MSCs on preventing gastrocnemius atrophy 6 weeks after implantation [11,24,25,28,29]. Among them, MSCs demonstrated beneficial effects in three studies but worsened muscle atrophy in the remaining two. The protective effects of MCSs on gastrocnemius atrophy 12 weeks after surgery were illustrated in five studies by three research teams, and four out of the five studies favored the MSCs treatment [24,25,29,36]. Meta-analysis indicated that MSCs improved gastrocnemius atrophy not after 6 weeks (difference in standard means (SMD), 95% confidence interval [15], fixed effect: 0.22 (−0.24 to 0.68), I^2^ = 54%, 86 rats) but after 12 weeks significant (difference in standard means (SMD), 95% confidence interval [15], fixed effect: 1.11 (0.61 to 1.6), I^2^ = 49%, 80 rats) after implantation (see Figure 6).

**Figure 7:**
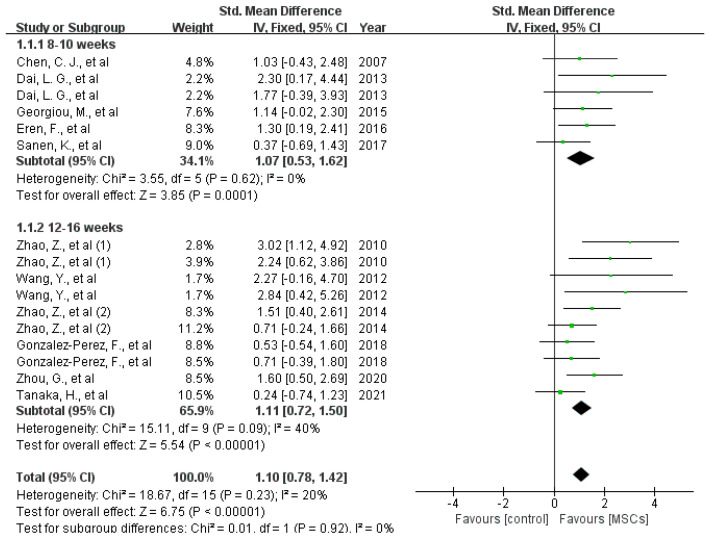
Meta-analysis of the effects of MSCs on axon number (middle and distal parts of the nerve defects) 8–10 weeks and 12–16 weeks after implantation [5,11,12,13,14,15,16,17,18,19,20,21,22,23,24,25,26,27,28,29,30,31,32,33,34,35,36]. Plots on the right-hand side indicate favoring MSCs or MSCs-loaded scaffolds, while plots on the left side indicate favoring placebo-control treatment or blank scaffolds.

**Figure 8:**
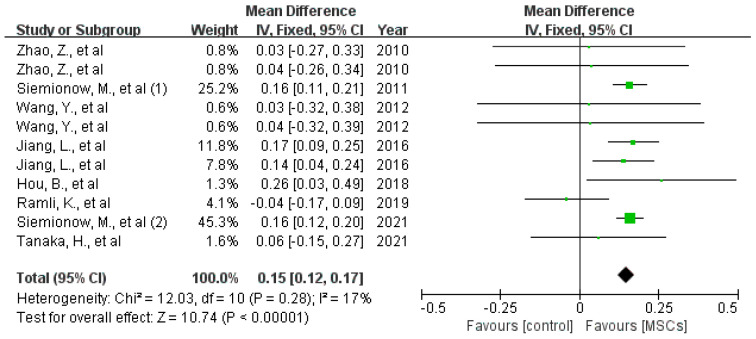
The forest plot shows the effect of MSCs on myelin thickness (middle and distal parts of the nerve defects) 12 weeks after surgery [5,11,12,13,14,15,16,17,18,19,20,21,22,23,24,25,26,27,28,29,30,31,32,33,34,35,36]. Plots on the left-hand side indicate favoring MSCs or MSCs-loaded scaffolds, while plots on the right side indicate favoring placebo-control treatment or blank scaffolds.

Histomorphometric assessment: The effect of MSCs on axon number and myelin thickness in the middle or distal portions of nerve defects was investigated in 11 studies, all of which indicated beneficial outcomes for MSCs. As shown in Figure 7, MSC treatment increased the axon number both at 8 to 10 weeks (difference in standard means (SMD), 95% confidence interval [15], fixed effect: 1.07 (0.53 to 1.62), I^2^ = 0%, 68 rats) and 10 to 16 weeks (difference in standard means (SMD), 95% confidence interval [15], fixed effect: 1.11 (0.72 to 1.50), I^2^ = 40%, 140 rats) after implantation [12,13,15,16,17,27,30,32,34,35,36]. Eight studies reported the effects of MSCs on myelin thickness 12 weeks after surgery. Meta-analysis indicated that the myelin sheath of MSC-treated rats was much thicker than in control animals (difference in means [38], 95% confidence interval [15], fixed effect: 0.15 (0.12 to 0.17), I^2^ = 17%, 129 rats) [5,18,20,26,29,30,32,35] (see Figure 8).

## 4. Discussion

This study aimed to systematically review the preclinical evidence for using MSCs to regenerate critical-sized peripheral nerve defects in experimental rats in vivo. Despite differences in donor species, delivery routes, and cell dosage, most included studies showed that the application of MSCs resulted in significantly better outcomes in terms of promoting nerve regeneration, improving nerve function, and preventing muscle atrophy. The SFI, conduction velocity, muscle weight, myelin thickness, and fiber count represent a variety of assessments for nerve regeneration, and for most of the assessed studies, MSCs were beneficial for these outcomes. However, a direct comparison between methods used in different studies is difficult due to the heterogeneity of outcome measures. From the five afore-listed outcome measures, only Zhou et al. (2020) used four in their results, Hou et al. (2018) and Tanaka et al. (2021) used three, and all other studies used two or fewer of the outcome measures assessed in this meta-analysis [18,30,36].

Looking at the studies individually, only four studies showed one outcome parameter with a tendency in favor of the control group without MSC application [26,28,29,36]. No study showed results favoring the control group in two or more assessed parameters. Thus, we can conclude a clear tendency across studies toward showing the beneficence of adjuvant MSC application. However, more comparable studies using the same methods and assessments are needed to isolate the effect of MSC use and decrease confounding effects such as the scaffold material. To further eliminate the potential influence of intrinsic factors, nine out of the 27 studies used decellularized nerve allografts to repair nerve defects [19,20,23,30,31,32,33,34,35]. However, across the board, we see a high variety in conduit materials as well as in MSC type, donor, and amount (Figure 2). This includes MSCs from bone marrow, adipose tissue, dental pulp, umbilical cord blood, and combinations of those from both human and rat donors. As conduits, different biological and synthetic materials from a variety of origins were used. This heterogeneity may suggest that there is a general positive effect of MSCs on the regeneration of critical-sized nerve gaps but as well poses a difficulty in comparing these results and finding the best solutions for future clinical application.

Clinically, critical gap-size nerve defects are defined as nerve defects of a size where a tensionless direct epineural coaptation of the proximal and distal nerve stump is not possible. However, when simulating them in the rat sciatic model, additional factors have to be taken into account. Rats have higher regenerative capabilities, and defect sizes cannot be scaled directly to the human body size [39]. Therefore, study results can be impacted by the high regenerative capacity of rodent peripheral nerves and not adequately represent effects due to the chosen gap-bridging method. In order to filter out studies eventually impacted by a limited gap size, the limit for inclusion was set at 15 mm, as described in the literature [40].

Injection of MSCs into injury areas without a scaffold might also promote nerve repair, but quite a number of MSCs will migrate out of the defect area. Nerve scaffolds can provide a space for MSCs to adhere to, prevent infiltration of surrounding tissues, and create a stable environment for nerve regeneration [41]. Generally, there are two main types of nerve scaffolds: autologous and artificial nerve scaffolds. Autologous nerve scaffolds possess a suitable regenerative capacity and excellent biocompatibility [42]. That is the reason why autologous nerve scaffolds are considered the gold standard for bridging peripheral nerve defects. However, there are also several drawbacks, including limited availability of donor nerves, complications at the donor site, increased risk of infection, and size mismatch between donor and recipient nerves [37,43].

In 10 studies, nerve scaffolds were manufactured with artificial biomaterials, including inorganic, organic macromolecular, and polymeric materials. Seventeen studies chose tissue-derived nerve scaffolds made of allogenic nerves, xenogeneic nerves, veins, or muscle. Despite these artificial nerve scaffolds having a noticeable difference in structure and composition from the physiologic nerve, they showed beneficial effects in all included studies. However, artificial nerve scaffolds also suffer from several limitations. For example, some materials, such as silicone-based nerve conduits, cannot be thoroughly degraded in vivo [44]. Furthermore, some organic macromolecular and polymeric materials’ metabolites may induce local inflammatory reactions [45].

MSCs have gained much attention in the field of tissue regeneration since they were first described in 1968 [46]. The understanding of how MSCs exert their beneficial effects during nerve repair has also evolved substantially over the past decades. Originally, it was suggested that MSCs promoted peripheral nerve regeneration mainly through differentiating into Schwann cells [47]. Later, studies showed that undifferentiated MSCs also possessed a powerful capacity to regenerate peripheral nerve injuries [12,33]. Sowa et al. found that transplantation of undifferentiated MSCs promoted regeneration of axons, formation of myelin, and restoration of denervation-associated muscle atrophy comparable to Schwann cell transplantation [48,49]. However, although MSCs have the potential to differentiate into Schwann cells, no implanted MSCs differentiated into Schwann cells at the end of the 4-week study [48,49]. These results suggest that the paracrine properties of MSCs essentially constitute the base for their medical applicability. It may explain why xenografted human MSCs also promoted the regeneration of the sciatic nerve and the recovery of its function in five studies included in this meta-analysis of preclinical studies.

MSCs were reported to produce a number of pro-regenerative agents for axonal regrowth, including brain-derived neurotrophic factor (BDNF), glial-cell line-derived neurotrophic factor (GDNF), and nerve growth factor (NGF) [50,51]. These proteins play essential roles in Schwann cell proliferation, survival, and differentiation. Moreover, exosomes from MSCs can mediate cell–cell communication by transferring miRNAs to recipient neurons to promote axonal growth, thus enhancing the neurogenesis and myelination of Schwann cells and neuronal cells [8,52]. Consistent with the above results, our meta-analysis revealed that SFI, nerve conduction velocity, gastrocnemius, and triceps surae muscle weights, axon numbers, and myelin sheath thickness were all increased in MSC-treated animals. Besides their neurotrophic functions, MSCs also improve the perilesional microenvironment by promoting vascularization and inhibiting inflammation. Previous studies showed high levels of vascular endothelial growth factor-A and angiopoietin-1 in condoned medium from MSCs [53,54]. Moreover, MSCs secreted a wide range of anti-inflammatory cytokines, including transforming growth factor (TGF)-β, IL-1Ra, and IL-10, and played a crucial role in regulating inflammation. In summary, MSCs modulate nerve regeneration through a quite complex mechanism, and further studies investigating the mechanisms of the beneficial effects of MCSs are still needed.

There is no doubt that MSCs have great potential in regenerative medicine. However, recent discoveries on the interaction of MSCs with tumors have generated doubts concerning the safety of their clinical application. MSCs possess inherent tumor-homing features and strong immunosuppressive abilities [55]. MSCs recruited by tumor cells can be activated by high concentrations of inflammatory cytokines within tumor microenvironments. Depending on the individual microenvironment of the tumor, their influence on the malignancy can have a wide range, from anti- to pro-tumorigenic. As a result, they may secrete a series of immunosuppressive factors, including indoleamine 2,3-dioxygenase, TGF-β, TNFα, IFNγ, prostaglandin E2, which can help tumor cells escape from immune surveillance [37]. Moreover, MSCs exposed to tumor microenvironments can be converted to cancer-associated fibroblasts, which promote angiogenesis and tumor growth through the secretion of stromal-cell-derived factor 1, VEGF, and TGF-β [56]. Although it is highly dependent on certain microenvironments and the signaling pathways leading to a pro-tumorgenic phenotype are not yet fully understood, the potential risk of tumor formation and aggravation should also be considered and thoroughly investigated when MSCs are used in the clinical setting.

Several limitations exist in the present study: (1) The dosage of MSCs varied considerably in different studies; the least amount used was 2.5 × 10^5^ cells, while the highest was 4 × 10^6^ cells. (2) Some studies preincubated MSCs with scaffolds in vitro for several days, while other studies directly implanted MCSs into the nerve defect, making it difficult to compare the dosage of MCSs in these studies. (3) Only a few studies indicated blinding of outcome assessors, and no studies described allocation concealment, blinding of the study team, random housing, and random outcome assessment. (4) Overall heterogeneity was moderate to high in the majority of analyses presented, which may hint at potential risks of publication or small study bias and differences in the effects observed. (5) Quantitative analysis was performed without correcting for possible confounders, such as MSC type and origin or scaffold type. (6) We excluded studies with a gap size of less than 15 mm, further reducing the number of overall included studies.

## 5. Conclusions

This metanalysis demonstrates the positive effects of additional MSC transplantation when bridging nerve defects in vivo studies. With the potential advantages and disadvantages of promoting tissue proliferation, further investigation is needed to ensure patient safety and enhance recovery after a peripheral nerve injury. Minding the general potential of this method, further research should focus on refining its different aspects, including the amount and origin of MSCs as well as the scaffold material, with comparable outcome measures to bring the use of adjuvant mesenchymal stem cells closer from bench to bedside.

## Figures and Tables

**Table 1:** Eligibility criteria for study identification according to the PICOS framework. In order to filter out studies eventually impacted by natural regeneration due to a limited gap size, the limit for inclusion was set at 15 mm.

	Inclusion Criteria
Population	Peripheral nerve injury over 15 mm in rats
Intervention	MSCs without genetic modification; MSCs-loaded scaffolds
Comparison	Sham; blank scaffolds
Outcome	Voluntary motor function, nerve conduction velocity, muscle atrophy, nerve fiber number, and myelin sheath thickness
Study design	Experimental animal study; English or Chinese language
Year of publication	No restriction

## Data Availability

The data presented in this study are available on request from the corresponding author. The data are not publicly available due to privacy reasons.
